# Supplementary material for: Psychometric Adaptation and Validity of the Resistance to Peer Influence Scale Among Young Chinese Drivers and Its Links with Peer Pressure and Risky Driving Behaviours
Source: Behav Sci (Basel). 2025 Sep 11;15(9):1237. doi: 10.3390/bs15091237 (PMC12466497; doi:10.3390/bs15091237)
Supplement: Supplementary file 1 [file behavsci-15-01237-s001.zip › behavsci-3827056-supplementary.pdf]

Supplementary Materials

Table S1. Normalized factor loading and item description (n = 269)

Item	Λ	M	SD
1. Some people go along with their friends just to keep their friends happy. BUT Other people refuse to go along with their friends want to do, even though they know it will make their friends unhappy.	0.628	2.56	0.86
2. Some people think it's more important to be an individual than to fit in with the crowd. BUT Other people think it is more important to fit in with the crowd than to stand out as an individual.	0.769	2.73	1.06
3. For some people, it's pretty easy for their friends to get them to change their mind. BUT For other people, it's pretty hard for their friends to get them to change their mind.	0.654	2.29	0.89
4. Some people would do something that they knew was wrong just to stay on their friends' good side. BUT Other people would not do something they knew was wrong just to stay on their friends' good side.	0.855	2.77	1.07
5. Some people hide their true opinion from their friends if they think their friends will make fun of them because of it. BUT Other people will say their true opinion in front of their friends, even if they know their friends will make fun of them because of it.	0.733	2.63	0.94
6. Some people will not break the law just because their friends say that they would. BUT Other people would break the law if their friends said that they would break it.	0.807	3.04	1.08
7. Some people take more risks when they are with their friends than they do when they are alone. BUT Other people act the same way when they are alone as they do when they are with their friends.	0.722	2.57	0.91
8. Some people change the way they act so much when they are with their friends that they wonder who they "really are". BUT Other people act just as risky when they are alone as when they are with their friends.	0.749	2.40	0.91
9. Some people say things they don't really believe because they think it will make their friends respect them more. BUT Other people would not say things they didn't really believe just to get their friends to respect them more.	0.727	2.65	0.91
10. Some people think it's better to be an individual even if people will be angry at you for going against the crowd. BUT Other people think it's better to go along with the crowd than to make people angry at you.	0.712	2.80	0.98
